# Biological Crusts to Increase Soil Carbon Sequestration: New Challenges in a New Environment

**DOI:** 10.3390/biology10111190

**Published:** 2021-11-16

**Authors:** Paola Duran, María de la Luz Mora, Francisco Matus, Patricio Javier Barra, Ignacio Jofré, Yakov Kuzyakov, Carolina Merino

**Affiliations:** 1 Biocontrol Research Laboratory, Universidad de La Frontera, Temuco 4780000, Chile; paola.duran@ufrontera.cl (P.D.); patricio.barra@ufrontera.cl (P.J.B.); 2 Scientific and Technological Bioresource Nucleus, Universidad de La Frontera, Temuco 4780000, Chile; mariluz.mora@ufrontera.cl; 3 Network for Extreme Environmental Research (NEXER), Universidad de La Frontera, Temuco 4780000, Chile; francisco.matus@ufrontera.cl; 4 Laboratory of Conservation and Dynamic of Volcanic Soils, Universidad de La Frontera, Temuco 4780000, Chile; ignacio.jofre@ufrontera.cl; 5 Division of Agricultural Soil Science, University of Göttingen, 37077 Göttingen, Germany; ykuzyakov@yandex.com; 6 Institute of Environmental Sciences, Kazan Federal University, 420049 Kazan, Russia

**Keywords:** biocrust functions, soil microbiota, CO_2_ mitigation, greenhouse gas (GHG) emissions, carbon balance, mineral weathering

## Abstract

**Simple Summary:**

Scientific knowledge should transcend the barriers between the laboratory and the field to act in the service of humanity. Considering the enormous potential that soil offers for organic carbon (SOC) sequestration for the mitigation of greenhouse gas (GHG) emissions, and considering the recognized ecological importance of biological soil crusts (biocrusts) to be applied in the soil–plant continuum, we propose three perspectives to apply biocrusts to sustainable agriculture.

**Abstract:**

The major priority of research in the present day is to conserve the environment by reducing GHG emissions. A proposed solution by an expert panel from 195 countries meeting at COP 21 was to increase global SOC stocks by 0.4% year^−1^ to compensate for GHG emissions, the ‘*4 per 1000′* agreement. In this context, the application of biocrusts is a promising framework with which to increase SOC and other soil functions in the soil–plant continuum. Despite the importance of biocrusts, their application to agriculture is limited due to: (1) competition with native microbiota, (2) difficulties in applying them on a large scale, (3) a lack of studies based on carbon (C) balance and suitable for model parameterization, and (4) a lack of studies evaluating the contribution of biocrust weathering to increase C sequestration. Considering these four challenges, we propose three perspectives for biocrust application: (1) natural microbiome engineering by a host plant, using biocrusts; (2) quantifying the contribution of biocrusts to C sequestration in soils; and (3) enhanced biocrust weathering to improve C sequestration. Thus, we focus this opinion article on new challenges by using the specialized microbiome of biocrusts to be applied in a new environment to counteract the negative effects of climate change.

## 1. Introduction

Among the main immediate challenges of humanity, the reduction in atmospheric CO_2_ concentrations and the emissions of other greenhouse gases (GHGs) is a major priority to counteract the negative effects of climate change [1,2]. Because soils can store two to three times more carbon (C) than the atmosphere, soil organic carbon (SOC) sequestration is a possible and efficient solution to diminish GHG emissions to the atmosphere [2]. In grasslands and agriculture, 47% of total potential mitigation arises from SOC protection and sequestration, while 20% involves other GHGs involved with improved soil management practices [3,4].

The enormous potential of soils to sequester C motivated the governments of 195 countries in 2015, convened in Paris for the 21st Conference of the Parties (COP 21), to launch a new global climate change agreement, the ‘*4 per 1000*′, with the main objective to increase global SOC stocks by 4 per 1000 (or 0.4%) per year as a compensation for global GHG emissions from anthropogenic sources (https://www.4p1000.org/, access on 1 August 2021). Despite the high aim of 4% per year being almost impossible, the development of strategies that increase C accumulation in soil should be a priority of governments, scientific communities, and modern agriculture [5].

In agriculture, many approaches have been focused on soil C regeneration through increased residue returns and biomass production (cover crops), and on decreasing C losses via reduced disturbance (no-till farming). These agronomic approaches, however, do not always produce net C gains since soil C accumulation is not a linear function of inputs [6]. One of the determinant keys of the C balance is the microbial mineralization of SOC, which results in CO_2_ losses [7]. Thus, the efficiency of soil microbes to process C is gaining interest as an important management strategy to increase SOC sequestration [6]. In this context, the interactions within biological soil crusts (biocrusts) are currently attracting scientific attention because of their ability to increase the fixation of nitrogen (N) and C, phosphorus (P) availability, and reduce nutrient leaching. Biocrusts are therefore considered as a key ecological microbial community in the continuity of soil biogeochemical cycles [8,9,10]. In order to obtain a closed cycle, all the different sources, cycling processes, and sinks need to be assessed by means of suitable methods, some of which will require a new approach.

### 1.1. Biocrust, a Photosynthetic Cell Factory for C Sequestration in Agriculture

Biocrusts cover about 12% of the Earth’s landmasses, thereby providing ecosystem services, affecting biogeochemical fluxes on a global scale, and strongly influencing soil–plant relationships; thus, facilitating edaphic engineering effects [11,12]. Biocrusts are found on almost all soil types, but are more commonly found in arid and cold regions, where plant coverage is low and more widely spaced. Across the globe, biocrusts can be found on all continents, including Antarctica [13,14].

**Figure 1:**
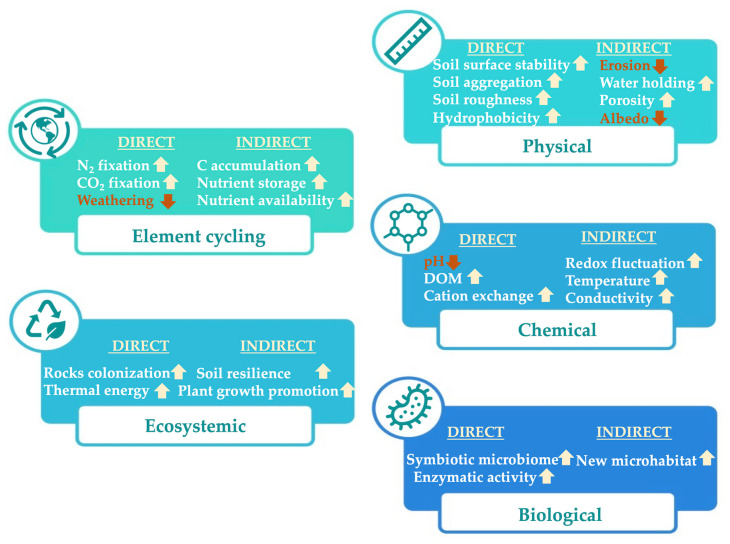
Effects of biocrusts on soil properties and element cycling. The direct and indirect effects are summarized according to their main mechanisms based on physical, chemical, biological, ecosystemic, and element cycling. The arrows before each point show an increase (↑) or decrease (↓) in the presence of biocrusts.

Biocrusts are formed by an association of soil mineral particles and micro-organisms, composed by free-living, lichenized, and mycorrhizal fungi, chemoheterotrophic bacteria, cyanobacteria, diazotrophic bacteria, archaea, eukaryotic algae, and bryophytes. Biocrusts can aggregate soil particles through excreting exopolysaccharides (EPSs), glycoproteins, and forming filament networks [15]. However, the species composition and physical appearance of biocrusts depend on the climate, soil properties, and disturbance conditions. For example, biocrusts are more dominated by green algae on more acidic and less salty soils. In contrast, cyanobacteria are more favored on alkaline soils [16]. Independent of climate, the abundance of lichens and mosses in biocrusts generally increases with increasing clay and silt content and decreasing sand (Table 1). Moist habitats generally support more lichens and mosses [17]. Due to their abilities, biocrusts are one of the major players of global C and N sequestration in soils. On the other hand, biocrusts also increase soil stability against erosion by forming aggregates, increasing porosity and water retention, leading to better seed germination [8,9,16,18]. Thus, an improved understanding of the structures, composition, and functions of biocrust microbiomes and their geological implication (geomicrobiology) allows changes in soil ecosystem structures to be forecast for long-term restoration due their essential roles related to physiological or chemical properties (Figure 1).

### 1.2. Biocrust, a Photosynthetic Cell Factory for C Sequestration in Agriculture

Baumann, Jung, Samolov, Lehnert, Büdel, Karsten, Bendix, Achilles, Schermer and Matus [16] evaluated the richness of green algae and cyanobacteria of biocrusts in four climate zones: arid, semi-arid, Mediterranean, and humid temperate. According to the morphological identification of the enrichment cultures, a total of twenty-four taxa of green algae and eighteen of cyanobacteria, regardless of climatic conditions, were found. Each biocrust was comprised of twelve to fifteen phototrophic species that used sunlight as an energy source to assimilate CO_2_, directly affecting the C cycle by C fixation [26]. Cyanobacteria are the oldest oxygenic photosynthetic organisms, producing O_2_ during photosynthesis as they fix CO_2_ dissolved in the water, thus having one of the most important metabolisms to have evolved on Earth [27,28]. The adaptation ability of cyanobacteria has allowed them to live in various conditions, including marine, freshwater, and terrestrial environments [29,30]. Cyanobacteria have been applied in medicine, cosmetic manufacturing, bioremediation, biofuel, and agriculture [29,31,32,33]. In fact, some filamentous cyanobacteria have evolved specialized cells to fix atmospheric N, known as heterocysts [34]. However, in recent years, more attention has been paid to their C sequestration potential [10,35]. For example, Kheirfam, Sadeghi and Darki [10] studied a factorial combination of bacteria and cyanobacteria from biocrusts and nutrients added to the field-collected soil. The authors showed that the inoculation of biocrusts had the potential to remove 0.85–1.07 g CO_2_ m^−2^ from the atmosphere.

Such scientific interest in the understanding of biocrusts’ contribution to global climate change advances the prediction, scaling, restoration, and C sequestration options that are at the forefront of contemporary biocrust science. In the present opinion manuscript, we discuss the structural, benefic, and evolutionary mechanisms to overcome environmental limitations by biocrusts. In addition, we suggest a new and revolutionary challenge for the new generation of bioinoculants based in biocrusts, such as host natural microbial engineering, where plants recruit their microbiome to increase C sequestration: new challenges in a new environment.

## 2. Limitations of the Broad-Scale Application of Biocrusts in Agriculture

**Figure 2:**
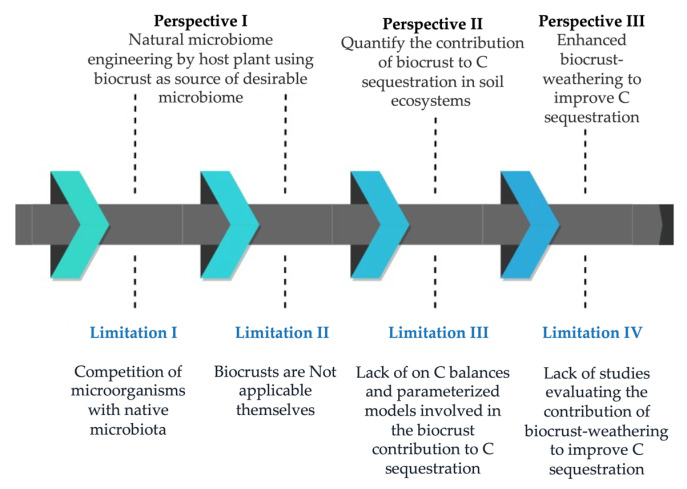
Proposal perspectives to solve practical limitations to the use of biocrusts for increased carbon sequestration.

Despite the demonstrated importance of biocrust-residing microorganisms to improve C sequestration, field applications are limited because (Figure 2):

(1) *Competition of micro-organisms (desirable strains from biocrusts) with native microbiota in a new environment*;

(2) *Biocrusts are not applicable themselves*;

(3) *A lack of parameterized C balance models simulating the contribution of biocrusts to the C sequestration processes*;

(4) *A lack of studies evaluating the contribution of biocrust weathering to improve C sequestration*.

## 3. Perspectives to Solve Practical Limitations of Biocrust Use for Carbon Sequestration

**Figure 3:**
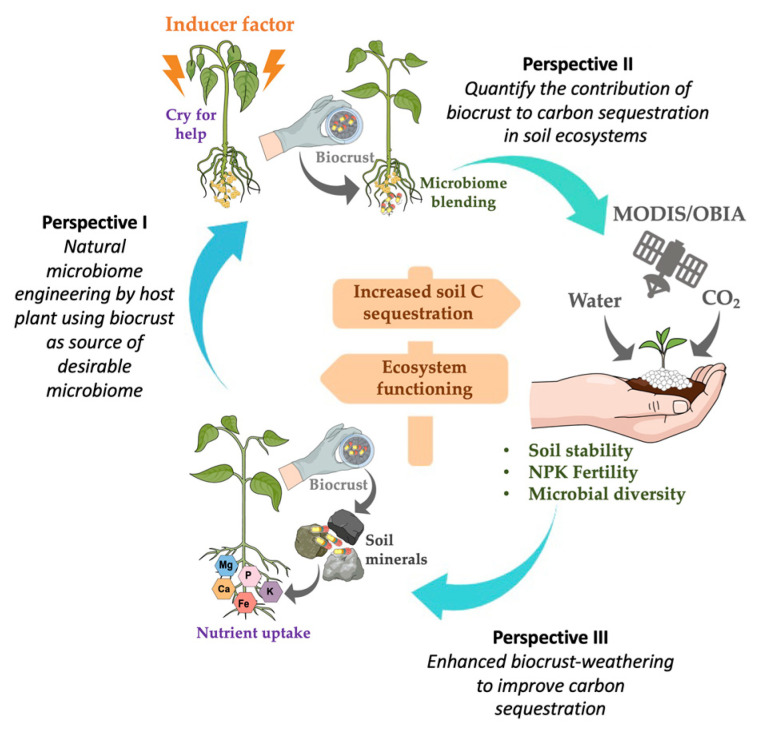
Proposal perspectives: biocrust applications in agriculture and in a new environment. The link between soil processes, microbiome, ecosystem services, weathering, and soil monitoring perspectives. The arrows indicate the relationship between soil–plant processes and biocrusts. The yellow arrow indicates the impact of the soil perspectives on regulating and provisioning ecosystem functioning and C sequestration.

Most research on biocrust inoculation has been conducted at a laboratory or on a plot scale, which is a necessary starting point; however, we need methods that can scale up the application, innovative methodologies, and treat ecological- and management-relevant areas. For these purposes, we propose three perspectives to implement effective biocrust application and monitoring to understand the main functions and services of the ecosystem (Figure 3).

### 3.1. Perspective I. Natural Microbiome Engineering by a Host Plant, Using a Biocrust as Source of Desirable Micro-Organisms

To decrease the first and second limitations, we suggest natural microbiome engineering by a host plant, using a biocrust as a source of a desirable microbiome. Host-mediated microbiota engineering (HMME) is a novel biological strategy that utilizes the intrinsic ability of plants to recruit and select their associated microbiome in their rhizobiome through cyclic differentiation and propagation [36,37]. HMME is a promising approach for improving host performance by engineering microbial communities for beneficial effects on growth, stress tolerance, and plant health [38,39]. This strategy enables the selection of a particular microbiome by visualizing changes in the host phenotype after several generations of growing the plant in the same place [40]. We recently report that the natural selection of microbiota over multiple generations can be induced through the existence of three factors: (1) a plant model, (2) an abiotic/biotic stressor or inducer factor, and (3) a desirable microbiome [41]. Thus, when a host plant is subjected to some type of abiotic/biotic stress or inducer factor, the plant can modify the composition of their exudates, resulting in the reassembly of the associated microbiomes, which in turn is reflected in modifications of the plant phenotype [42,43,44], entailing an adaptation of the host plant [42,45]. These ‘selected’ micro-organisms would have an advantage over external microbiota, and the reassembled microbiome can optimize the plant’s response against stress or an inducer factor [46,47]. Bakker, et al. [48] suggested the concept of ‘*cry for help*’, wherein the stage of an outbreak of biotic or abiotic disease plants recruit protective microbiota, mainly by the exudation of photo-synthetically fixed C in the rhizobiome and favoring endosphere colonization [49,50]. Thus, plants are able to recruit a specialized microbiota when subjected to an adverse condition.

We propose the use of HMME using a (i) *model plant*, which could be a representative grassland species, (ii) subjected to *elevated CO_2_* (eCO_2_) as an inducer factor, due to eCO_2_ influencing the richness, composition, and structure of soil microbial community to C sequestration [51], (iii) grown in soil mixed with a biocrust (*desirable microbiome*). After some growth cycles under these eCO_2_ conditions, plants (themselves) could select those micro-organisms that give them a comparative advantage in C sequestration (induced by eCO_2_). These micro-organisms can be horizontally transferred among cycles and vertically to descending generations by seeds. From this, is possible to obtain bioinoculants by culturable micro-organisms or whole microbiota by using rhizobiome extracts (Figure 3). For example, Jochum and collaborators induced drought tolerance after six rounds of HMME selection, and core microbiota functionality was transferable in a subsequent assay [36]. Another study induced earlier or later flowering times in *Arabidopsis thaliana* plants using HMME selection [37]. Recently, HMME was also applied in *Nasonia*; the insects were exposed over successive generations to subtoxic levels of atrazine, and changes were observed in the structure and function of the gut microbiome that conveyed that microbiome-mediated atrazine resistance was inherited and increased over successive generations of *Nasonia vitripennis* [44]. This innovative biotechnology allowed for the endophytic bioinoculant (inside the roots) naturally selected by the host plant to be obtained, which avoids competition with native microbiota (***limitation one***) and is applicable under field conditions because it could be directly applied to seeds (***limitation two***).

### 3.2. Perspective II. Quantify the Contribution of Biocrusts to Carbon Sequestration in Soils

The monitoring of C fluxes in terrestrial ecosystems to accurately quantify and predict C balance is, globally, one of the highest research priorities [52]. These predictions are essential for identifying and quantifying sinks and sources of greenhouse gas (GHG, e.g., CO_2_) emissions (***limitation three***). Research about biocrusts related to the C cycle has been carried out mainly under controlled conditions with temperate forest soils, agricultural ecosystems, and drylands. However, field-scale studies are scarce [19]. Quantifying the C stocks and fluxes in these biomes and determining the processes that regulate them are crucial for a basic understanding of the C cycling of these ecosystems that cover 45% of the Earth’s land area [53]. It is estimated that increasing soil C reserves in these low-productive ecosystems could significantly reduce atmospheric CO_2_ levels [54], considering that soil C uptake estimates are 3.5 to 5.2 Gt year^−1^ [55].

The C budgets of these forest and agriculture ecosystems can be increased with the application of a biocrust. However, we must also consider how microbial compositions affect the responses of the C flux to abiotic conditions [56,57], since each taxon may have a different response and could form different interactions among them (***limitation one***). Quantifying the magnitude of the contribution of each species or functional group to the ecosystem C balance will improve estimates of C budgets in these ecosystems. A biocrust can contribute differently to C storage: (a) aggregating soil particles through the secretion of exopolysaccharides, forming networks of filaments that increase the stability of the soil against erosion and other degradation factors [58,59]; (b) increasing porosity [60]; (c) retaining water and/or infiltrating it [61,62]; (d) increasing soil fertility by accumulating nutrients [27,59]; and (e) helping in the establishment of other organisms such as mosses, lichens, cyanobacterias, micro-fungi, and plants, increasing the storage potential of C [63]. These functions performed by a biocrust are relevant since it is one of the predominant soil covers, covering up to 70% of the surface in some areas (e.g., dryland), and it is the primary source of soil organic carbon (SOC) in many of them. It is estimated that biocrusts represent ~15% of global terrestrial C stock and ~40–85% of N fixation worldwide [64,65]. Therefore, quantifying C fluxes and nutrients through the assembly of biocrust communities under field conditions would significantly enhance our understanding and prediction, through mathematical models, of how specific pressures derived from global change could alter the structure of the biocrust community and, accordingly, guide our efforts towards enhancing C sequestration and nutrient availability in soils [66,67].

With this goal, we suggest that it is important to consider: (i) in situ monitoring of CO_2_ and other GHG fluxes with the microbiota derived from an inoculated biocrust from perspective I in the soil and to relate these fluxes with environmental factors and physico-chemical properties that control GHG emissions; (ii) a second significant contribution to knowledge would be to map the distribution of biocrust types (in successional stages) in the basins of various ecosystems using ground-based remote sensing techniques. As low- and moderate-resolution satellite imagery (e.g., Landsat and Moderate Resolution Imaging Spectroradiometer (MODIS)) or object-based image analysis (OBIA) approaches are often used for mapping with very-high-resolution imagery when the pixel resolution is inadequate.; (iii) all contributions (balance) on C fluxes (both biocrusts and micro-organisms) under natural field conditions to include them in model-based soil C monitoring systems; and (iv) possible associations between specific groups of micro-organisms below the biocrust, enzymatic activities involved in the C, N, and P turnover, and soil physico-chemical variables (e.g., TOC, TN, P, pH, and carbonates). Improving the production of reliable maps of biocrust cover further depends on the availability of imaging systems which provide not only adequate spatial and spectral resolution but are also capable of collecting images sufficiently frequently.

### 3.3. Perspective III. Enhanced Biocrust Weathering to Improve Carbon Sequestration

Enhanced mineral weathering is a C sequestration process that could remove more than 2 billion tons of CO_2_ each year. Silicate minerals exposed to the weathering surface can sequester atmospheric CO_2_ and transform it into HCO_3_^-^, thereby reducing the intensity of atmospheric greenhouse effects. However, it is a process that usually takes thousands of years. Even so, rock weathering is an important component to consider for geological carbon sinks. Carbon sinks derived from carbonate weathering and silicate weathering are the two primary mechanisms underlying rock weathering carbon sinks [68]. Unfortunately, the time this process takes is too long to compensate for CO_2_ flux from human activities. This limitation can be compensated through an inoculated biocrust from perspectives I and II to increase weathering rates. Because when the biocrust comes into contact with the rock, it triggers a chemical process known as the Urey reaction. This reaction removes CO_2_ from the atmosphere and combines it with water and calcium or magnesium silicates, leaving the CO_2_ trapped in these carbonates in the soil. C capture’s accelerated chemical weathering process could remove more than 2 billion tons of CO_2_ each year [65]. This is because approximately 95% of the Earth’s crust is made of silicate minerals, which are silicon and oxygen compounds.

Exemplarily, the chemical reaction can be followed through the dissolution of anorthite (Equation (1)). The dissolution of primary silicates leads to secondary precipitates, releasing cations, and transforming CO_2_ into HCO_3_:

*Ca*_2_*Al*_2_*Si*_2_*O*_8_ + 2*CO*_2_ + 3*H*_2_*O* → *Al*_2_*Si*_2_*O*_5_(*OH*)_4_ + *Ca*_2_^+^ + 2*HCO*_3_ (1)

If supersaturation concerning individual carbonate phases is reached, solid carbonates might form (Equation (2)):

*Ca*_2_+ + 2*HCO*_3_^−^ → *CaCO*_3_ + *CO*_2_ + *H*_2_*O* (2)

Carbonate formation is an important mechanism for the in situ fixation of CO_2_ through carbon capture and storage [69]. However, enhanced weathering aims to convert CO_2_ into alkalinity, as the formation of carbonates will reduce the process’s efficiency (Equation (2)). The maximum CO_2_ amount drawn from the atmosphere through silicate dissolution is a function of the cation flux (mostly Ca_2_^+^, Mg_2_^+^, K^+^, and Na^+^); which is charge-balanced by HCO_3_^−^ formation. In addition, CO_2_ fixation through the dissolution is based on Al conservation through the formation of secondary minerals (Equation (1)). However, the dissolution of the aluminosilicate can precede the formation of the secondary phase [70] (Equation (3)), and far from equilibrium conditions can be sustained during basalt weathering, for instance, through the complexation of Al_3_^+^ with organic acids [71]:

*Ca*_2_*Al*_2_*Si*_2_*O*_8_ + 8*CO*_2_ + 4*H*_2_*O* → 2*Al*_3_^+^ +*Ca*_2+_ + 8*HCO*_3_^−^ + 2*SiO*_2_(*aq*) (3)

It is essential to consider that the investigations of the patterns, mechanisms, and rates of weathering with biocrusts are in their infancy (**limitation four**). What we know comes largely from geomicrobial interactions studied in similar settings, that are endolytically within rocks. Because of its importance in global carbon cycling, many scientists have carried out research on silicate rock weathering carbon sink and made progress toward understanding the related mechanisms. In this sense, Chen, et al. [72] analyzed the effects of biocrust incorporation on the soil, showing that it enriched the soil with biogenic elements (C, N, and P) and generated the leaching of many metals and metalloids from the mineral phase. This effect on the rock is achieved through the reactivity of the biocrust metabolic products (e.g., extracellular polymeric substances) excreted by the micro-organisms on the mineral surface, acidification or decrease in the redox potential due to the permanent coating of minerals by exopolymeric substances, and the secretion of specific metallic ligands and other organic complexes [73,74]. Moreover, in the rock fragments (because of weathering), more Mg, Fe, and Ca silicates are exposed, increasing porosity and permeability [75,76]. Additionally, the reactive mineral fraction (Fe and Mn) forming associations with the organic matter has been suggested as another C sequestration strategy to mitigate climate change [77,78,79]. The combination and intensity of these mechanisms in biocrusts likely vary, but microbial stabilization is determining in all of them, and abiotic factors have not been examined in much detail. In this sense, carrying out studies about the relationships between natural chemical weathering rates and controlling parameters (e.g., temperature, precipitation, and pH) can help to clarify the discrepancy observed between field and laboratory data [80,81] and could allow the up-scaling of local measurements to a global scale (perspective II), contributing to the refinement of global CO_2_ consumption estimations. Future research can help to elucidate the generality of biocrust responses to the suite of global changes with which they are faced and increase our understanding of the mechanisms that drive this change. Studying the parameters controlling weathering rates associated with biocrust in natural settings can improve the feedback between climate and weathering and its role in the short-term carbon cycle and climate change [82,83,84].

The responses of biocrusts to climate change appear to be particularly strong and, while different biocrust organisms will respond differently to changing climatic conditions, the data suggest that increasing temperatures and altered precipitation patterns, as well as strong interactions between the two, are significantly modifying the structure, function, and resilience of biocrust communities. Thus, depending on how microbial community profiles change, there may be more implications for mineralization and organic C storage [65]. Unfortunately, studies on the relationship between biocrusts and weathering are few and appear only to account for current research. Thus, we propose an intensive study of the relationship between the effects of micro-organisms derived from biocrusts (perspective I) on mineral weathering, soil formation, and continuous C sequestration (perspective II) to validate the advantage and attributes of biocrusts. Consequently, we postulate that the exhaustive study of the composition and dynamics of biocrust and their interactions with the physico-chemical properties of soils under the new prevailing conditions as a result of the atmospheric increase in GHGs has enormous potential to be used as a biotechnological tool to increase the sequestration of C in soils.

## 4. Conclusions

Investigations of biocrust weathering patterns, mechanisms, and rates are in their infancy. Most research on biocrust inoculation has been conducted at a laboratory, which is a necessary starting point. However, we need to scale-up the research using common protocols in relevant areas to land managers, and bridge the gap between science and practice. The next frontiers for biocrusts need to better document how biological (e.g., species composition/function and organism condition) and physical factors (e.g., activity rates and times as determined by climatic factors, soils) influence C fixation and loss. Across all scales, we need to understand and observe biocrust photosynthesis and respiration, and what portion of C losses is due to other sources, such as bacteria, fungi, and soil carbonates by indirect inoculation, where host plants recruit specific microbiomes in the rhizobiome to induce C sequestration. Future research can help elucidate the generality of biocrust responses to the suite of global changes they face and increase our understanding of the mechanisms that drive this change. For this, we proposed three perspectives to solve practical limitations to using biocrusts to increase carbon sequestration: utilizing the natural microbiome of a host plant, quantifying the contribution of biocrusts to carbon sequestration in soil ecosystems, and enhanced biocrust weathering to increase the level of nutrients released and carbon sequestration, addressing the spatial relationships between biocrusts and ecological processes and quantifying their contribution to ecosystem functionality at local to landscape scales.

## Figures and Tables

**Table 1:** Preference of biocrust-composing microbial groups to climatic and edaphic conditions (nd = not determined).

Organism Group	Climate	Soils	References
Cyanobacteria	Semi-arid cold	Alkaline, loamy clay soil	[9]
	Dry sub-humid coastal area	Nd *	[18]
	Arid	Nd	[16]
	Semi-arid	Nd	[16]
	Semi-arid	Sandy loams	[8]
	Mediterranean	Nd	[19]
	Humid	Nd	[19]
	Semi-arid	Oligotrophic	[20]
Cyanobacteria/lichens	Cold desert	shrub interspaces	[21]
Cyanobacteria/moss	Cold desert	beneath *Artemisia tridentata*	[21]
Bacteria (diazotrophs and chemoheterotrophs)	Semi-arid	Oligotrophic	[20]
	Cold desert	Burnt soils	[21]
	Semi-arid cold	Alkaline, loamy clay sil	[10]
	Cold desert	Burnt soils	[21]
Green algae	Arid	Nd	[16]
	Semi-arid	Nd	
	Mediterranean	Nd	
	Humid	Nd	
Lichens	Mediterranean semi-arid	nd	[22]
	Semi-arid to dry sub-humid	Arenitic	[23]
		Dolomitic	
		Arenic fluvisols	
		Leptic chernozems	
		Luvic phaeozems	
		Calcaric cambisols/regosols/leptosols	
	Semi-arid	nd	[24]
Mosses	Semi-arid; arid	nd	[22,25]
	Semi-arid to dry sub-humid	Arenitic	[23]
		Luvic litosols	
		Leptic chernozems	
		Litosols	
		Leptic chernozems	
		Litosols	
		Dolomitic	
		Calcic luvisols; humic regosols	
		Arenic fluvisolsLeptic chernozems	
		Luvic phaeozems	
		Calcaric cambisols/regosols/leptosols	
		Calcaric regosols	

* Not determined.

## Data Availability

Not applicable.
